# Resection of ruptured hepatic teratoma in an adult

**DOI:** 10.1016/j.ijscr.2018.11.032

**Published:** 2018-11-22

**Authors:** Jonathan Ramkumar, Andrew Best, Ananta Gurung, Anne-Marie Dufresne, George Melich, Elena Vikis, Shawn MacKenzie

**Affiliations:** a Department of General Surgery, Royal Columbian Hospital, University of British Columbia, New Westminster, BC, Canada; b Department of Radiology, Royal Columbian Hospital, University of British Columbia, New Westminster, BC, Canada; c Department of Pathology, Royal Columbian Hospital, University of British Columbia, New Westminster, BC, Canada

**Keywords:** Case report, Teratoma, Hepatic teratoma, Hepatobiliary, Surgery, General surgery

## Abstract

Introduction: Extragonadal locations of teratomas are uncommonly reported in the literature. Teratomas are neoplasms usually found in the gonadal organs: ovaries and testis. The majority of teratomas are found in the pediatric age group. Furthermore, teratomas originating in the liver are exceedingly rare with only 11 case reports in adult populations.

Presentation of case: We present a case of a 65 year-old female who presented to hospital with sudden onset abdominal pain from a centrally located ruptured hepatic teratoma on CT scan. The patient underwent urgent surgery. The diagnosis of cystic mature teratoma was confirmed on histopathology. Patient was discharged on post-operative day 5. At 12 week follow-up, no post-operative complications were identified.

Discussion: Hepatic teratomas are a rarely encountered neoplasm, especially in the adult population. Our case report is unique, as it represents the only clinical presentation of mass rupture in an adult liver teratoma. CT scan identified a well circumscribed mass containing adipose tissue, fluid, and calcifications characteristic of teratoma. Complete surgical resection is mainstay treatment. A definitive diagnosis of a mature teratoma is confirmed by histopathological findings.

Conclusion: Presented is a rare case of ruptured hepatic teratoma in an adult who underwent surgical resection.

## Highlights

• Rare presentation of ruptured hepatic teratoma in an adult.
• <1% of teratomas are found to originate from liver and predominantly found in pediatric populations.
• Complete surgical resection is mainstay treatment.
• Definitive diagnosis is found on histopathological results.

## 1 Introduction

Teratomas are rare germ cell tumors occurring most commonly in the gonadal organs. Etymologically, the word teratoma is derived from the Greek word “teratos” which means monster [1]. By definition, teratomas are derived from 2 or more germ cell layers: ectoderm, mesoderm, and endoderm [2]. Teratomas present most commonly in the ovaries and testes, followed rarely in the anterior mediastinum, retroperitoneum, sacrococcygeal region and cranium [3]. Rarely, reported are gastrointestinal tract and liver teratomas which comprise less than 1% of all teratomas [[4], [5], [6]]. The majority of liver teratomas are seen in children under 3 years of age, reflecting the origin from primordial germ cells [7]. Therefore, we present an exceedingly rare adult case of a ruptured mature hepatic teratoma for surgical resection. This case is reported in line with the SCARE criteria [8].

## 2 Presentation of case

A 65-year-old Punjabi-speaking female with treated hypocholesteremia presented to the emergency department with sudden onset right upper quadrant pain. The patient had not had any previous surgeries, denied any smoking, alcohol or recreational drug use, and did not remark any medical conditions in her past family history. Of note, on her immigration examinations a decade early, she was told of a small abnormality on her liver. No management or follow-up was recommended at that time. On physical exam, she had some fullness and mild abdominal tenderness to right upper quadrant with no diffuse peritoneal signs.

**Fig. 1:**
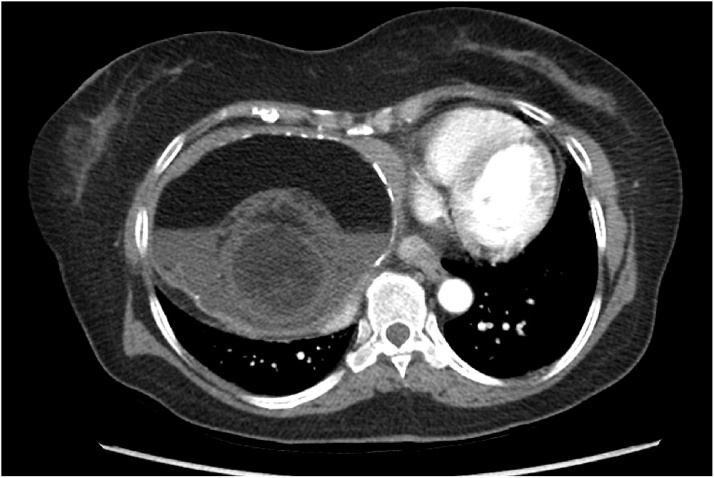
CT Abdomen/Pelvis showing axial section of CT a large mass in the right upper quadrant measuring approximately 15 × 12 × 11 cm. A conspicuous fat fluid level is present, as well as partial rim calcification.

Initial contrast enhanced CT imaging scan demonstrated a large mass in the right upper quadrant measuring approximately 15 × 12 x 11 cm. A conspicuous fat fluid level is present, as well as partial rim calcification (Fig. 1). There was no vascular enhancement to any component of the mass. As is often the case with such large lesions, identifying the site of anatomic origin was challenging; the right hepatic lobe or the porta hepatis were offered as possibilities based on imaging. Hepatic parenchyma was not convincingly seen along its superior margin, and there was no continuity with the stomach or pancreas.

**Fig. 2:**
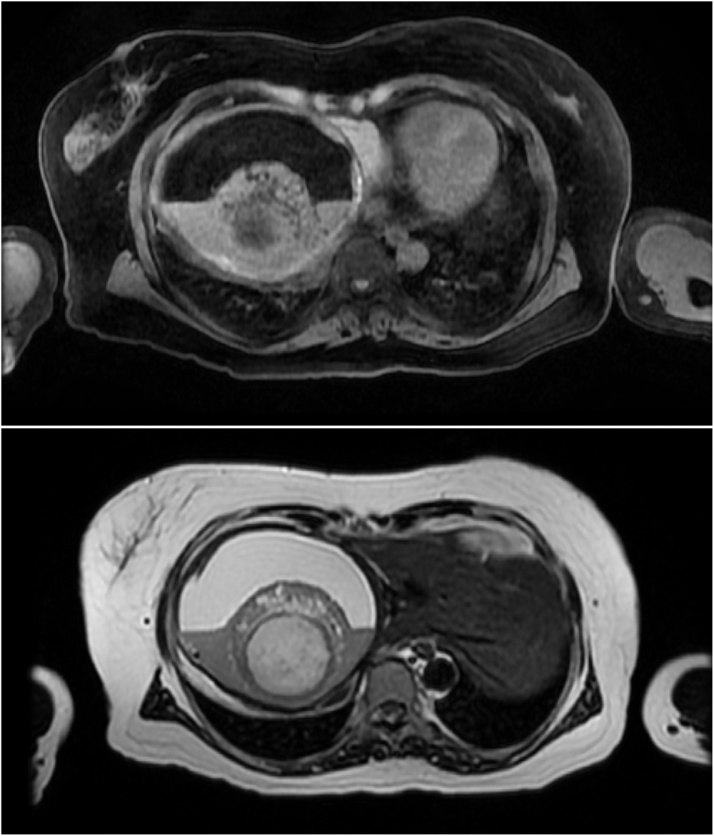
MRI Abdomen/Pelvis T1/T2 signal axial section of right upper quadrant mass with presence of fat fluid level and no internal enhancement.

**Fig. 3:**
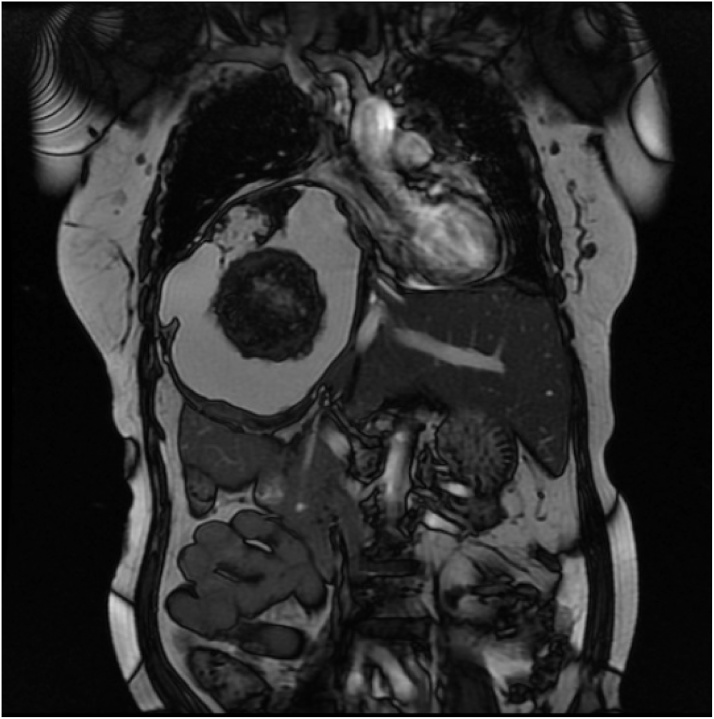
MRI Abdomen/Pelvis Coronal section of right upper quadrant mass demonstrating mass effect and marked upward displacement of right diaphragm.

Multisequence contrast-enhanced MRI imaging again demonstrated the presence of a fat fluid level (Fig. 2), and the absence of internal enhancement. There was marked upward displacement of the right hemidiaphragm as the result of mass effect (Fig. 3). Although the location was very unusual, the imaging appearances were characteristic for a dermoid/teratoma. The remainder of the liver, aside from distortion related to the mass effect, was unremarkable. A small amount of perihepatic fluid was seen on imaging suggesting tumor rupture. Additional pelvic MRI imaging demonstrated normal appearing ovaries for patient age.

Based on a tentative diagnosis of teratoma and evidence of acute rupture of the mass, surgical intervention was recommended after patient was treated with fluid resuscitation and broad-spectrum antibiotics. A preoperative anesthetic consult was obtained and she was brought for surgery with a hepatobiliary surgeon. The patient underwent a laparotomy and partial hepatectomy. Intraoperatively, the tumor was found to be unresectable with R0 intent. The tumor demonstrated involvement the bifurcation of the porta hepatis and the retroperitoneum, including the retro-hepatic inferior vena cava. Based on the fact that the tumor previously ruptured, the anterior wall of the tumor was resected removing liver segments 5, 8 and a portion of 4, thus debulking the tumor by removing 95% of the tumor volume. The small amount of remaining tumor remained on the left and right biliary pedicles. Intraoperatively, the tumor contained thick sebaceous liquid and debris. The patient experienced no intraoperative complications with an estimated blood loss of 100 cc.

**Fig. 4:**
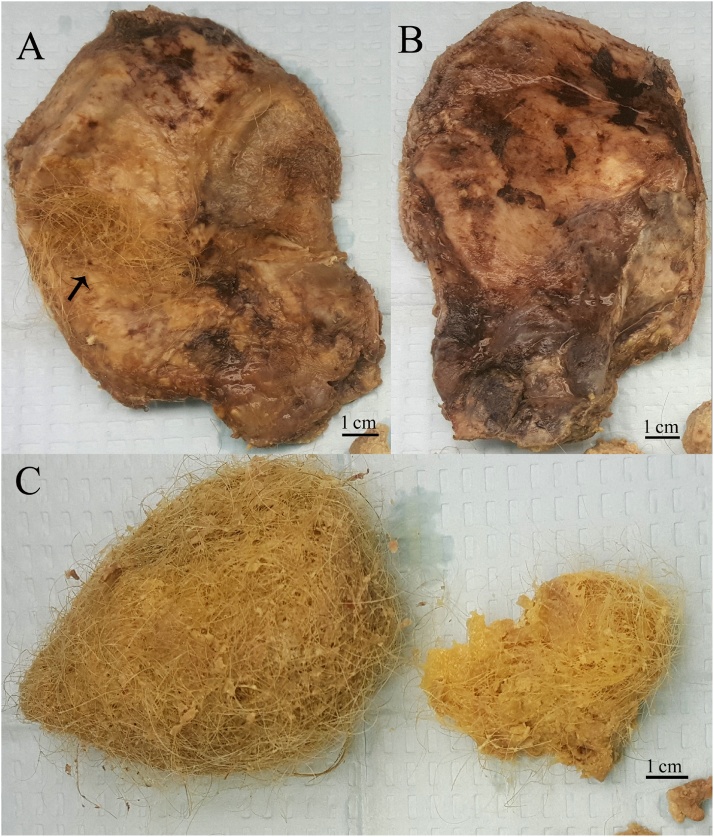
The internal aspect of the cyst (A) contained adherent hair (arrow) while the external aspect (B) demonstrated areas of necrosis, hemorrhage and fibrinopurulent exudates. Several detached fragments of cyst contents consisted of conglomerates of hair admixed with soft, caseous, necrotic material (C).

Gross pathological examination revealed a firm rubbery textured cyst wall (13 × 9 x 0.8 cm) and cyst contents (Fig. 4). Multiple strands of hair were adherent and embedded with the internal aspect of the cyst wall. Patchy foci of necrosis, hemorrhage and fibrinopurulent exudates were noted on both the internal and external aspects. The cyst wall was serially sectioned to reveal a fibrous and hyalinized cut tissue. Normal liver parenchyma was not appreciated. The remaining tissue consisted of a conglomerates of hair (reminiscent of gastric bezoars) admixed with soft caseous material.

**Fig. 5:**
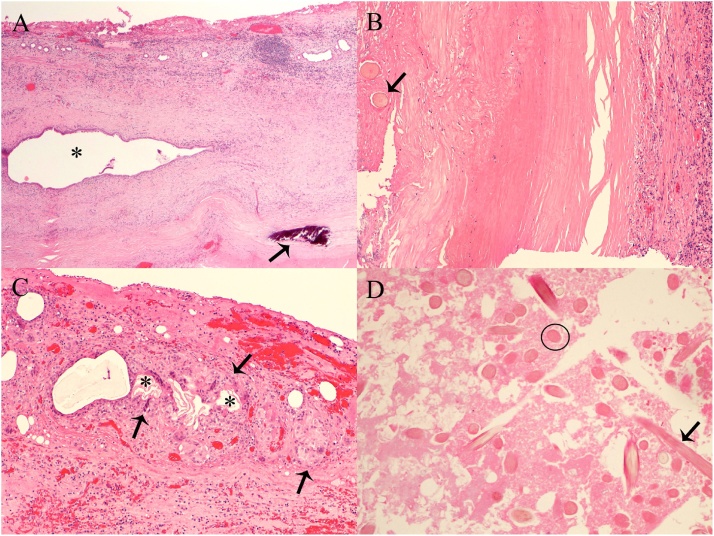
Histological examination revealed hyalinized cyst wall containing distended bile ducts (asterisk) and areas of dystrophic calcification (arrow, A, 40X). Numerous hair shafts within the cyst wall were identified (arrow, B, 100X) as well as foreign body giant cells (arrows, C) surrounding fragments of keratin (asterisks, C, 100X). Sections from the cyst content showed hair shafts cut transversely (circle) and longitudinally (arrow) with surrounding necrotic material (D, 200X). All sections stained with hematoxylin and eosin.

Microscopic exam of the cyst wall demonstrated extensive areas of hyalinization with areas of dystrophic calcification (Fig. 5). Cystically dilated spaces lined by bland cuboidal to columnar biliary epithelium were identified. Embedded within the cyst wall were hair shafts and fragments of keratin surrounded by foreign body giant cells. Representative sections from the caseous cyst contents revealed numerous hair shafts and necrotic debris. Immature neuroepithelial tissue was not identified. Based on the pathological findings, a diagnosis of mature cystic teratoma was rendered. We postulated that the findings represented a monodermal mature cystic teratoma of ectodermal origin given the presence of hair. An alternate explanation possibility includes a conventional mature cystic teratoma (containing at least two germ cell layers) in which the other elements were not identified given the burnt out nature of the lesion. Given that the tumour was debulked, it is entirely possible that the other germ cell layers were not represented in the resected specimen.

There were no significant acute post-operative issues and patient was discharge home safely on post-operative day 5. There were no postoperative complications at 6 weeks during follow-up visit. A follow-up CT scan was obtained which showed status post partial hepatectomy with thickened tissue along the bile duct at the porta hepatis, consistent with residual teratoma cyst wall.

## 3 Discussion

Hepatic teratomas are a rarely encounter tumor with less than 30 case reports can be found in the literature and the majority in pediatric populations [[4], [5], [6], [7], [8], [9]]. Only 11 of these have been present in adult population in English literature with the largest tumor recorded as 27 cm in diameter [10]. Our case report is unique as it represents the only clinical presentation of mass rupture in an adult liver teratoma [10].

Primordial germ cells follow a midline path and descend into the pelvis as ovarian and testicular cells, which explain their more common midline and paramedian locations. It is hypothesized, during the organogenesis period, arrest of these germ cells in migration along this path can lead to teratoma formation in extragonadal locations [3,5]. Hepatic teratomas range from asymptomatic and incidentally found on CT scans to presenting with symptoms related to mass effect such as abdominal distension, fullness, nausea and vomiting [5,6]. Our patient presented with acute onset of right upper quadrant abdominal pain, likely due to the rupture of the teratoma.

A CT scan showing a well circumscribed mass containing adipose tissue, fluid and calcifications is characteristic of the radiologic findings of teratomas [2,11,12]. As well, CT scan can determine the mass effect of the tumor if present. Teratomas need to be differentiated from other benign and malignant fat-containing liver masses [12]. In our case report, the CT scan showed a central containing soft tissue and complex fluid filled mass with peripheral calcifications with parenchymal disruption due to the perforation. Complete surgical resection remains the best treatment option [13,14]. At the time of surgery, the sebaceous fluid was found outside the lesion in the right upper quadrant sub-diaphragmatic space representing tumor rupture. Unfortunately, with tumor involvement of porta hepatis and rupture, complete resection (R0 intent) of the tumor was not possible. Therefore, a debulking procedure resecting greater than 95% of the mass was performed.

A definitive diagnosis of a mature teratoma is confirmed by histopathological examination [6]. Microscopic examination allows the categorization of tumors as benign or malignant, and allows assessment for the presence of immature and mature elements. Features which are important for prognostication [5,13]. Histopathological examination of our patient’s specimen revealed a benign monodermal mature cystic teratoma of ectodermal origin given the presence of hair. Teratomas are characterized by teeth, hair follicles, and sebaceous contents which is consistent with our case report [13].

## 4 Conclusion

In summary, we present an exceedingly rare clinical presentation of an acute hepatic teratoma rupture in an adult patient who underwent near complete 95% surgical resection. Clinical work-up includes a CT scan, with confirmation of diagnosis of hepatic teratoma on histopathology. Resection remains the mainstay of treatment. This case adds to the limited literature of the patient presentation, clinical work-up, and management liver teratomas.

## Conflicts of interest

None.

## Funding

None.

## Ethical approval

Not applicable for case reports as per journal policy.

## Consent

Written informed consent was obtained from the patient for publication of this case report and the accompanying images. A copy of the written consent is available for review by the Editor-in-Chief of this journal on request.

## Author contribution

Jonathan Ramkumar: drafting of the article, critical revision of the article for important intellectual and clinical content.

Andrew Best: image creation, critical revision of the article for important intellectual and clinical content.

Ananta Gurung: image creation, critical revision of the article for important intellectual and clinical content.

Anne-Marie Dufresne: critical revision of the article for important intellectual and clinical content.

Elena Vikis: critical revision of the article for important intellectual and clinical content.

George Melich: critical revision of the article for important intellectual and clinical content.

Shawn MacKenzie: drafting of the article, critical revision of the article for important intellectual and clinical content, final approval of the version to be submitted.

## Registration of research studies

Not applicable.

## Guarantor

Shawn MacKenzie.

## Provenance and peer review

Not commissioned, externally peer reviewed.
